# Supplementary material for: Antimicrobial Resistance in Enterococcus spp. Isolates from Red Foxes (Vulpes vulpes) in Latvia
Source: Antibiotics (Basel). 2024 Jan 24;13(2):114. doi: 10.3390/antibiotics13020114 (PMC10885957; doi:10.3390/antibiotics13020114)
Supplement: Supplementary file 1 [file antibiotics-13-00114-s001.zip › Supplementary_Table S1_Isolates_included_in_the _present_study_major revision.pdf]

Table S1.

***Enterococcus* spp. isolates included in the present study**

Sample code	Origin of sample - region	Origin of sample - parish	*Enterococcus* species	No. of identified resistance	Phenotypical resistance pattern	WGS analysis (ENA accession)	MLST ST (gdh, gyd, pstS, gki, aroE, xpt, yqiL for *E. faecialis* or atpA, ddl, gdh, purK, gyd, pstS, adk for E. *faecium*)
423602	Gulbenes nov	Beļavas pag	*Enterococcus faecium*	0	0	Included (ERX11781920)	603 (4, 2, 12. 9. 1. 7. 5)
429441	Gulbenes nov	Beļavas pag	*Enterococcus faecium*	1	Q/D	Included (ERX11781937)	N (n, 3, 1. 2, 1, 1, 1)
	Gulbenes nov	Beļavas pag	*Enterococcus faecalis*	1	TET	Included (ERX11781941)	498 (29, 6, 30, 5, 60, 1, 4)
433769	Augšdaugavas nov	Vecsalienas pag	*Enterococcus faecalis*	0	0	Included (ERX11781931)	116 (17, 2, 22, 1, 14, 14, 1)
433771	Augšdaugavas nov	Kalupes pag	*Enterococcus faecium*	1	TET	Included (ERX11781929)	832 (60, 3, 1, 2, 2, 1, 1)
3802	Rēzeknes nov	Lendžu pag	*Enterococcus faecalis*	0	0	Excluded, did not reach the threshold of >90% target genes (ERX11781911)	482 (14, 6, 11, 45, 4, 20, 28)
3808	Rēzeknes nov	Feimaņu pag	*Enterococcus faecium*	1	ERY	Included (ERX11781912)	N (n, 8, 8, 8, 11, 10, 6)
2124	Alūksnes nov	Jaunlaicenes pag	*Enterococcus faecium*	0	0	Included (ERX11781909)	N (2, 3, 1. 2, 1, n, 1)
17285	Talsu nov	Lībagu pag	*Enterococcus faecalis*	0	0	Included (ERX11781913)	950 (99, 2, 30, 3, 21, 1, 7)
426383	Ludzas nov	Isnaudas pag	*Enterococcus faecium*	1	TET	Included (ERX11781925)	22 (2, 3, 1, 2, 1, 1, 1)
428642/1	Rēzeknes nov	Maltas pag	*Enterococcus faecium*	1	DAP	Included (ERX11781932)	32 (3, 3, 1, 2, 1, 1, 1)
424290	Balvu nov	Šķilbēnu pag	*Enterococcus faecium*	0	0	Included (ERX11781921)	N (2, 40, 12, 3, 1, n, 1)
426804	Rēzeknes nov	Mākoņkalna pag	*Enterococcus faecium*	1	CIP	Included (ERX11781926)	29 (4, 5, 1, 3, 1, 1, 1)
428644	Rēzeknes nov	Maltas pag	*Enterococcus faecium*	1	CIP	Included (ERX11781927)	822 (4, 5, 1, 3, 1, 7, 1)

427166/2	Preiļu nov	Galēnu pag	*Enterococcus durans*	1	TET	Included (ERX11781922)	NA
427167/2	Preiļu nov	Galēnu pag	*Enterococcus faecalis*	0	0	Included (ERX11781939)	16 (5, 1, 1, 3, 7, 7, 6)
422861	Rēzeknes nov	Feimaņu pag	*Enterococcus faecalis*	0	0	Included (ERX11781923)	749 (90, 1, 18, 31, 39, 20, 88)
23152	Tukuma nov	Smārdes pag	*Enterococcus faecium*	1	ERY	Included (ERX11781918)	60 (13, 8, 8, 8, 11, 10, 6)
23065	Ventspils	Vārves pag	*Enterococcus faecium*	0	0	Included (ERX11781917)	2019 (13, 13, 18, 17, 11, 19, 6)
3829	Rēzeknes nov	Audriņu pag	*Enterococcus faecium*	0	0	Included (ERX11781915)	32 (3, 3, 1, 2, 1, 1, 1)
30266	Ventspils nov	Puzes pag	*Enterococcus hirae*	1	DAP	Excluded, low coverage (<30) (ERX11781928)	NA
2123	Alūksnes nov	Jaunlaicenes pag	*Enterococcus faecium*	1	TET	Included (ERX11781908)	1863 (5, 3, 6, 6, 6, 1, 1)
3821	Jēkabpils nov	Zasas pag	*Enterococcus faecium*	0	0	Included (ERX11781914)	1045 (51, 2, 12, 9, 1, 1, 5)
2110	Gulbenes nov	Galdauskas pag	*Enterococcus faecalis*	0	0	Included (ERX11781910)	1265 (12, 32, 3, 17, 6, 2, 72)
424211/2	Preiļu nov	Saunas pag	*Enterococcus faecium*	2	TET-ERY	Included (ERX11781930)	NEW (109, 16, 8, 72, 2, 69, 15)
424365	Rēzeknes nov	Feimaņu pag	*Enterococcus hirae*	1	TI	Included (ERX11781933)	NA
421603/1	Augšdaugavas nov	Biķernieku pag	*Enterococcus durans*	1	TI	Included (ERX11781934)	NA
423118/1	Augšdaugavas nov	Dvietes pag	*Enterococcus faecalis*	0	0	Included (ERX11781935)	NEW (8, 7, 7, 5, 4, 4, 5)
423522	Alūksnes nov	Jaunlaicenes pag	*Enterococcus durans*	0	0	Included (ERX11781936)	NA
420362	Ludzas nov	Ciblas pag	*Enterococcus faecium*	0	0	Included (ERX11781919)	52 (12, 9, 6, 3, 1, 13, 1)
418636	Balvu nov	Lazdukalna pag	*Enterococcus faecalis*	0	0	Included (ERX11781916)	719 (12, 32, 32, 51, 6, 20, 5)
427166/1	Preiļu nov	Galēnu pag	*Enterococcus faecalis*	0	0	Excluded, low coverage (<30) (ERX11781938)	NA, previously excluded, because of the low coverage

427175/3	Preiļu nov	Saunas pag	*Enterococcus faecalis*	0	0	Included (ERX11781940)	NEW (96, 2, 37, 1, 76, 15, 11)
426073	Balvu nov	Šķilbēnu pag	*Enterococcus faecalis*	0	0	Included (ERX11781924)	86 (6, 10, 11, 17, 4, 2, 1)

Q/D – quinupristin/dalfopristin, TET – tetracycline, ERY – erythromycin, DAP – daptomycin, CIP – ciprofloxacin, TI- tigecycline, N – no value, NA – not applicable
